# Isolation and identification of resveratrol-producing endophytes from wine grape Cabernet Sauvignon

**DOI:** 10.1186/s40064-016-2571-0

**Published:** 2016-07-08

**Authors:** Ya Liu, Lijun Nan, Junchao Liu, Haiyan Yan, Dianpeng Zhang, Xinnian Han

**Affiliations:** College of Food, Shihezi University, Shihezi, 832000 People’s Republic of China; Institute of Plant and Environment Protection, Beijing Academy of Agriculture and Forestry Sciences, Beijing, 100097 People’s Republic of China; Institute of Crops, Xinjiang Academy of Agriculture and Reclamation Science, Shihezi, 832000 People’s Republic of China

**Keywords:** Cabernet Sauvignon, Endophytes, Identification, Resveratrol, Screening

## Abstract

**Objectives:**

Obtain endophyte strains with effective resveratrol production from superior grapevine variety Cabernet Sauvignon in Xinjiang and determine related taxonomic position of the strain.

**Results:**

Seventy-three strains of endophytes, including 23 strains of bacteria, 14 ones of actinomycetes, 24 fungus and 12 yeasts, were isolated, respectively. The distribution law of endophytes was spring (30.14 %) = summer (30.14 %) < autumn (39.73 %) in different seasons, while the fruit (12.33 %) < leaf (20.55 %) < stem (32.88 %) < root (34.25 %) in different tissues and organs. From the 36 strains of endophytic fungi isolated, seven strains producing polyphenols were screened by ferric chloride–potassium ferricyanide color reaction. C2J6, stable genetic properties producing highly 1.48 mg L^−1^ of resveratrol, was identified as *Aspergillus niger* by 26S rDNA-ITS sequence analysis after thin-layer chromatography sieve analysis, ultra violet wavelength scanning and high performance liquid chromatography, respectively.

**Conclusions:**

There were the certain number and kinds of endophytes in the various tissues of Cabernet Sauvignon, which, to a certain extent, reflected the biological diversity of plant endophytes. The fact that the fungus C2J6 producing resveratrol in grape was acquired attested the special ability of the endophytes to produce the same or similar bioactive substances as the host plants.

## Background

Endophytic bacteria, actinomycetes and fungi, important components of plant micro-ecological system, were systematically distributed in the tissue, organs or cell gap of the roots, stems, leaves, seeds and fruits (Stone et al. 2000; Azevedo et al. 2000). In collaboration with the long evolution, the endophytes and their host plants formed a mutually beneficial relationship *between each other* (Saikkonen et al. 2004; Aly et al. 2011). Endophytes cound not only participate in synthesis or transformation of the plant secondary metabolites, but also form a large number of secondary metabolites which possessed biological active functions and great potential applications in the field of medicine, health and agriculture (Strobel and Daisy 2003; Aly et al. 2010). Hundreds of species of plants were found in the rich diversity of the endophytic fungi resources, from which a large proportion of novel structures and unique active compounds were screened and discovered (Strobel et al. 2004; Rodriguez et al. 2009). Some endophytes generated many compounds with similar physiological activities to the host secondary metabolites, such as terpenoids, alkaloids, saponins, sterols, quinones, indole, amines, peptides and polyphenol, as well as with biological activities, like antitumor, antibacterial, insecticide, immunosuppression and antioxidant (Strobel 2003; Guo et al. 2008; Chandra 2012; Gutierrez et al. 2012).

It had been verified that endophytes, such as *Pestalotiopsis guepini* and *Seimatoantlerium tepuiense*, could generate antitumor bioactive substances alcohol (Heinig and Jennewein 2009); while endophytic fungus *Phomopsis cassiae* could acquire Du Song *Eremophilane sesquiterpenes* 3,11,12-trihydroxycadalene which had strong antibacterial activity (Silva et al. 2006); the endophytic fungus *Chaetomella* sp. from *Eucommia ulmoides* was able to create flavonoids (Yao and Wei 2011); the *Curvularia* endophytes of *Ocotea corymbosa* plant was capable of emerging benzopyran (Teles et al. 2005); the endophytes of *Murraya paniculata* obtained alkaloids such as Spiroquinazoline alkaloid (Huang et al. 2007); stemona root endophytic bacteria *Paenibacillus polymyxa* achieved an extracellular polysaccharide (Liu et al. 2009). In addition, Terminalia morobensis *Pestalotiopsis microspora* endophytic fungi fermentation fluid in the two novel compounds *pestacin* and isopestacin (Strobel et al. 2002; Harper et al. 2003), as well as mangrove endophytic fungus *Alternaria* sp. also produced new secondary metabolites, like 10-oxo-10H-phenaleno [1,2,3-de] chromene-2-carboxylic acids I and xanalteric acids II with antioxidant activities (Kjer et al. 2009). According to reports, 51 % of the new bioactive substances recently discovered were from endophytes, and only 38 % came from soil microbes (Hyde and Soytong 2008). From the point, the endophytes of plant provided abundant resources for natural products.

Resveratrol, a stilbene polyphenol substances from natural secondary metabolites of plants (Burns et al. 2002; Poltronieri et al. 2013), often found in grapes, peanuts, Polygonum cuspidatum, veratrum, or cassia seed, had a large number of beneficial medical functions to human body, such as antiinflammatory, antiallergic, antitumor, regulating blood fat and anti pathogenic microorganism. Therefore, the ingredient had been widely applied in some fields, including health food, medicine and cosmetics (King et al. 2006; Jianrui et al. 2007; Poltronieri et al. 2013).

The preparation methods of resveratrol covered mainly natural plant extraction and chemical synthesis. The former method had limited sources of raw materials, and resveratrol yield was greatly affected by variety, season, climate, habitat and other factors. However, the synthesis technology of the later was characterized by the method complex (more than nine step methods), time-consuming, lower yield (15 %) and security problems, of which must be also considered ultimately.

There had been widely popular that the microbial fermentation produced scores of the biological activity materials, such as antibiotics, vitamins and hormones, depending on its many advantages with the cultivated and controlled fast and easily, the high yield and the low cost, without limitation by the seasons, climate and geographical constraints, and also improving the strains characteristics by means of mutation breeding. Therefore, this method had become one of the most effective ways to acquire the natural bioactive substances resveratrol.

Shihezi city, “gold zone” for the winegrapes cultivation, is located in the Northern Piedmont of the Tianshan Mountains and the Southern Margin of the Junggar Basin (85°–86°30′E, 43°30′–45°40′N). The Cabernet Sauvignon is always the main varieties of wine grapes in Shihezi area, and the resveratrol content in Cabernet Sauvignon dry red wine was more than 1 mg L^−1^. Based on the fact, the endophytes had the ability to synthesize the same or similar physiological active substances as host plants do, our objective is to find an endophytic fungi producing resveratrol in Cabernet Sauvignon.

## Methods

### Sampling of grapevines

Wine grape Cabernet Sauvignon, 5 years old, was collected at the junction of the third zone and fourth zone in Zhangyu Chateau in Shihezi city on a sunny day without wind at 8:00–10:00 on April 25, July 8 and September 20, 2011, respectively. The separation materials collected, the root, stem, leaf and fruit of Cabernet Sauvignon (the root, stem and leaf collected only on April 25), were immediately transported to the laboratory with the sterile bag at 4 °C, respectively. The materials were immediately rinsed with aseptic water on a sterile operating table to remove the dust, dirt and some microorganisms on the sample surface. After drying at room temperature, the root, stem, leaf and fruits collected were preserved at 4 °C, respectively.

### The surface sterilization of the samples and the pure culture of the endophytes

The samples above were soaked by 75 % alcohol for 3 min before washing with sterile water and drying using filter paper, respectively, and then treated by 10 % effective chlorine of sodium hypochlorite for 20 s prior to drying employing filter paper again, and finally rinsed by aseptic water for 5 times. Following slice of 5–8 mm long segment in term of the phloem of the root and stem, a 5–8 mm × 5–8 mm of the tablet as for the leaf, and 5 × 5 × 5 mm of diamonds without the fruit peel for the sarcocarp under the aseptic operation, which were laid upon the surface of the culture medium and pressed slightly to better integration between the culture and the sample, after the sterilizing surface and peeling epidermi, respectively. The samples placed on the middle of each Petri dish, keeping a certain distance of 4–5 blocks, were cultured under the suitable temperature. The three parallel Petri dishes were prepared for each tissue. The colonies cultivated were picked out and transferred carefully to a new sterile plate for re-culturing when the new hyphae or fungi culture appeared. After repeating the procedure several times, the endophytes of pure culture in different grapevine organs were just obtained.

### Medium and chemical agents

The potato dextrose agar (PDA), the separation and purification culture media of endophytic fungi, was prepared as follows: the 200 g of fresh potato filtrate through four layers of gauze, after boiling for 30 min, was added to 1000 mL with supple water, and supplemented 20 g of the filtrate agar and 20 g of glucose, respectively. Then the mixture, adjusted to pH 7.0, was subjected to 121 °C high pressure steam sterilization for 20 min. Agar was not added in the fermentation liquid of PDA. Gaus No. l medium acting as the isolation and purification culture of endophytic actinomycete was prepared according to the method described by Cao et al. (2004), and nutrient broth–yeast extract medium employed for the isolation and purification culture of the endophytic bacteria was also made up according to the method of Zinniel et al. (2002).

Standard resveratrol was purchased from Beijing century Aoke Biological Technology Co. Ltd. While the chemical agents as analytical reagents, such as methanol, acetonitrile, beef extract, peptone, agar, soluble starch, sodium hypochlorite, ethanol, glucose, sodium chloride, KNO_3_, K_2_HPO_4_, MgSO_4_·7H_2_O, FeSO_4_·7H_2_O, ethyl acetate, ferric chloride, potassium ferricyanide, toluene, acetic acid, and ethyl acetate, were provided by Beijing Baiyi Innovation Technology Co. Ltd.

### Preliminary screening of resveratrol-producing endophytes

Endophytic fungi purified were inoculated to 250 mL of Erlenmeyer flask including 100 mL liquid medium, respectively, and incubated at 115 rpm for 3 days at 28 °C simultaneously. Then the fermentation liquid was centrifuged at 4000 rpm for 10 min. Preliminary screening of chromogenic reaction: the endophytic fungus producing resveratrol could be screened according to the resveratrol property with ferric chloride–potassium ferricyanide color reaction. While the color reaction of the fermentation liquor was as follows: the mixture, 0.1 % FeCl_3_: 0.1 % K_3_[Fe(CN)_6_] = 1:1 (v/v), was served as chromogenic agent. 2 mL of coarse extraction liquid per sample concentrated above was mixed with the same volume of methanol, and then added two drops of chromogenic agent, meanwhile the color changes were recorded, respectively. The polyphenols containing resveratrol would be indicated by the indication of the blue color.

### Re-screening of resveratrol-producing endophytes

#### Thin layer chromatography (TLC)

The steps of TLC analysizing resveratrol qualitatively included:

(A) Ten microlitre of the strains fermentation liquid above after the centrifugation and 5 μg mL^−1^ resveratrol standard solution was spotted into the same silica gel plate (20 × 20 cm) in chromatography cylinder, then expanded vertically upward with the function of the developing solvent (toluene: ethyl acetate: acetic acid = 15:3:1, v/v), respectively. The thin layer plate was immediately removed and blowed dry by blower after the developing solvent launched to 1 cm of location close to the top of the thin layer plate.
(B) After the silica gel plate above was sprayed uniformly by the color developer, 0.1 % FeCl_3_: 0.1 % K_3_[Fe(CN)_6_] = 1: 1 (v/v) and dried, the distance was measured immediately between the blue spot center and origin as well as between the origin and solvent. Finally, the R_f_ value (rate of flow or retention factor) was calculated. The R_f_ value is a constant for a given component under the same experimental conditions. The R_f_ value may be calculated from the following equation. The R_f_ value itself is unitless.
  $$R_{f} = \tfrac{a}{b}$$
  where a is distance of the center of the sample spot from the origin; b is distance of the solvent front from the origin.

#### Extraction, separation and purification of resveratrol in the fermented liquid

Extraction and separation of crude resveratrol was carried out by a slight modification of the method of Zeng et al. (2012).

Thirty-six strains of endophytic fungi purified were inoculated to 250 mL of Erlenmeyer flask including 100 mL liquid medium, respectively, and incubated at 115 rpm for 3 days at 28 °C simultaneously. Then the collected supernatant of fermentation liquid centrifuged at 4000 rpm for 10 min, after vacuum concentration at 60 °C, was extracted 3 times by 50 mL of ethyl acetate finally. The raffinate in the ethyl acetate layer were collected and washed three times by 3 % (v/v) of NaHCO_3_ solution, before concentrated under the vacuum at 50 °C the extract liquor discarded NaHCO_3_ solution layer were dehydrated by the anhydrous magnesium sulfate and filtered by filter paper, then blowed dry by pure nitrogen, dissolved by 2.0 mL of methanol, and filtered by 0.45 μm of microporous membrane, successively, thus crude resveratrol products were obtained.

Purification of resveratrol in the crude products: the method of Jiang (2008) and Cai (2010) could be used in the trial by a slight modification. The steps were as follows: (1) crude extract of resveratrol was preliminarily purified and separated by AB-8 type macroporous adsorption resin; (2) 0.5–1.0 mg mL^−1^ of the samples were desorbed by 70 % ethanol (pH 7) at the rate of l mL min^−1^; (3) the samples isolated preliminarily was detached with the eluent of chloroform: methanol (15:1, v/v) after separating with silica gel chromatography, and the final residual liquid dried by nitrogen gas after vacuum concentration at 50 °C was just purified resveratrol, with a total process recovery above 77.3 %, of more than 90 % purity.

#### Ultraviolet wavelength scanning

Five milligram of purified resveratrol sample, weighed accurately, was diluted with aseptic water to the scale and shook well after completely dissolved by a small amount of methanol in a 50 mL of clean brown volumetric flask, which was 0.10 mg mL^−1^ of the resveratrol solution. Afterwards, 5 mL of accurate resveratrol solution, diluted with methanol to the scale in 25 mL of volumetric flask, was scanned by ultraviolet/visible spectroscopic measurements UV-1700 (Shimadzu) in 200–500 nm of the spectral range with methanol as CK. Thus the maximum absorption wavelength could be obtained from the absorption peak of the corresponding spectrum. It was obvious that the resveratrol existed in the extract liquid when the maximum absorption was at 306 nm.

#### Quantitative analysis of resveratrol by liquid chromatography (LC)

The methods of Zeng et al. (2012) and Standards Press of China (2006) were referred in the trial.

(A) Preparation of resveratrol standard solution (0.5, 1, 2.5, 5, 10, 15 and 20 μg mL^−1^). Firstly, 5 mg of the resveratrol standard weighed accurately was dissolved into 50 μg mL^−1^ of standard stock solution with the methanol. Secondly, 0.1, 0.2, 0.5, 1.0, 2.0, 3.0 and 4.0 mL of the standard stock solution from the above were precisely drawn and put into a 10 mL volumetric flask, respectively. Then, shaken well, sealed, stored after it was set with methanol to constant volume and finally, they were analyzed in order.
(B) Chromatographic condition: liquid chromatography (LC-2010 A HT type, Shimadzu) was accepted. A two-element of gradient elution protocols was employed on Waters XTeera MS C18 (4.6 × 250 mm, 5 μm) chromatographic column: 5 % acetonitrile in 95 % distilled water for the first 5.0 min; 60 % acetonitrile in 40 % distilled water for the second 28 min; 85 % acetonitrile in 15 % distilled water for the further 33 min; 5 % acetonitrile in 95 % distilled water for the final 40 min. The column temperature, flow rate and ultraviolet wavelength were 35 °C, 0.2 mL min^−1^ and 306 nm, respectively. The injection volume was 10 μL. Under such chromatographic conditions, resveratrol was eluted, and basically achieved baseline separation; retention time was 22.696 min.
(C) Drawing of standard curve: the resveratrol standard solutions above, triplicate per concentration, were detected according to (B), respectively. Therefore, the regression equation of the resveratrol, Y = 9 × 10^−6^X + 0.298 (R^2^ = 0.9993), based on the regression curve according to the integral value of peak area (Y) as ordinate and solution concentration (X, g L^−1^) of the standard sample as the abscissa, showed good linear relationship between 0.5 and 20 g L^−1^.
(D) Determination of resveratrol content: resveratrol purified was separated from the sample and diluted after dissolved with methanol to 25 mL, 1.0 mL of which was filtrated to automatical sample vials with 0.22 μm filter membrane, and 10 μL of the final filtrate was injected in LC before detection. The chromatographic peak of resveratrol in the samples was qualified by the retention time, meanwhile the resveratrol content was calculated through external standard method based on the peak area of the samples.

### Genetic stability of the resveratrol production

C2J6 was grown in PDA medium with shaking (150 rpm, 28 °C) for 3 days. The content of resveratrol produced by C2J6 was detected by LC as described above, and genetic stability of the resveratrol production was determined by successively subculturing five generations strain in PDA.

### Identification of resveratrol-producing endophytes

The resveratrol-producing endophytes were identified using morphological characteristics and molecular tools. The experiments of the morphological identification were carried out as described by Kim and Baek (2011). The strain was identified by sequencing the internal transcribed spacer 1 (ITS1), 5.8S ribosomal RNA gene and internal transcribed spacer 2 (ITS2) according to White et al. (1990) and the D1/D2 domain at the 5′ end of the LSU rRNA gene according to Kurtzman and Robnett (1998). The DNA from fungi cell suspensions grown in YPD for 48 h was extracted employing NucleoMag 96 Plant Kit (Macherey-Nagel, Oensingen, Switzerland) and Kingfisher magnetic particle processor (Thermo Labsystems, Basingstoke, UK) following the manufacturers’ protocols. The ITS regions were amplified by means of genomic DNA as a template and universal primers ITS1 and ITS4, while the D1/D2 domains were augmented through the primers NL-1 and NL-4 on the genomic DNA.

Twenty microlitre of PCR contained 1 µL DNA template (50 ng), 200 mM of each deoxynucleotide triphosphate, 2 µL of tenfold buffer (*Taq* DNA Polymerase, Qiagen, Chatsworth, CA, USA), 0.7 mM each primer, and 1.0 U *Taq* DNA Polymerase (Qiagen).

PCR program for ITS regions followed: 95 °C, 3 min; 34 cycles; 94 °C, 15 s; 55 °C, 45 s; 72 °C, 55 s; 72 °C, 7 min. Meanwhile, the program for D1/D2 domain was: 95 °C, 10 min; 30 cycles: 94 °C, 30 s; 55 °C 30 s; 72 °C, 45 s; 72 °C, 7 min. A 10 µL aliquot of PCR products from each reaction, electrophoresed in TBE buffer including 2.0 % agarose gel, was stained with SYBR SAFE (Invitrogen, Eugene, OR, USA). Gel images were finally acquired with a Gel Doc 1000 System (Bio-Rad Laboratories, Hercules, CA, USA). PCR amplification products were cloned into the PCR4 TOPO vector (Invitrogen) using the TOPO TA cloning kit following the manufacturer protocol and sequenced by Zhong-mei-Tai-he Sequencing Company (Beijing, China) and an Illumina HiSeq-2000 Sequencer (Illumina, USA).

## Results and discussion

### Isolation and purification culture of grapevines endophytes

As could be seen in Table 1, 73 strains of endophytes were obtained from grapevines collected in Shihezi city. While Zeng et al. (2012) acquired only 30 endophytes from skin, spike stalk and stalk of mature wine grape “Melot” collected in Shaanxi in August, which demonstrated that the strain number of endophytes in Shihezi area was more than that in Shaanxi one. In the trial, four sorts of endophytic strains, including the 24 strains of endophytic fungi, 12 strains of endophytic yeast, 14 strains of endophytic actinomycetes and 23 strains of endophytic bacteria, were isolated from grapevines, and the numbers relationship among them were: yeast (16.44 %) < actinomycetes (19.18 %) < bacteria (31.51 %) < fungi (32.87 %). Furthermore, the rates relationship of endophytic fungi between the different organ were as follows: root (34.25 %) > stem (32.88 %) > leaf (20.55 %) > fruit (12.33 %), while the proportional relationship among the endophytic fungis isolated during the different season was spring (30.14 %) = summer (30.14 %) < autumn (39.73 %). Therefore, the value of the endophytic microbes varied with the different varieties, different tissues and different seasons, which reflected an abundant biodiversity of endophytes.

**Table 1:** The endophytes distribution from different tissues of grapevine during different seasons

	Root (number)	Stem (number)	Leaf (number)	Fruit (number)	Number (number)	Ratio (%)
Spring						
Bacteria	–	7	–	–	7	9.59
Yeasts	2	1	1	–	4	5.48
Fungi	3	1	4	–	8	10.96
Actinomycetes	2	1	–	–	3	4.11
Summer						
Bacteria	3	4	2	2	11	15.07
Yeasts	2	1	–	–	3	4.11
Fungi	2	1	–	2	5	6.85
Actinomycetes	2	1	–	–	3	4.11
Autumn						
Bacteria	–	2	2	1	5	6.85
Yeasts	2	–	–	3	5	6.85
Fungi	2	3	5	1	11	15.07
Actinomycetes	5	2	1	–	8	10.96
Total number	25	24	15	9	73	100
Ratio (%)	34.25	32.88	20.55	12.33	100	

– That no strain has been isolated

Compared to the research reported on other plants, the number of the endophytes obtained in this research were relatively small, which was maybe explained by the several possible reasons: (1) the thorough disinfection to the samples surface killed some beneficial endophytic microbes; (2) the growth of the endophytes was obviously adjusted by the ecological environment during the periods; (3) some endophytic fungi could not be isolated or even cultured with the current methods. Therefore, we should strictly control the disinfection time of the material surface and the concentration of the disinfectant, and maintain the original ecological environment keeping the survival of the endophytic microbes as far as possible in the experiment to ensure the representative sample from the isolated endogenous microbes.

### Pre-screening the resveratrol-producing endophytes from grapevines

The studies had shown that the resveratrol was a kind of the flavonoid polyphenol compounds carrying stilbene type structure, possessed the characteristics of the phenol and stilbene, and appeared the blue under the ferric chloride and potassium ferricyanide solution. Therefore, the effective method of the characteristics reaction could employ the preliminary qualitative analyses of the resveratrol (Bavaresco et al. 2012; Díaz et al. 2012).

The fermentation liquids including 36 strains of endophytic fungi were ensured with color reaction, respectively. The color gamut, from the yellow green, light blue and light blue to the dark blue, was observed during the whole color reaction. As a result, the fermentation liquid of a-total-16-strains revealed the blue reaction, among which some strains including C2J6, C2Y6, C1G2, XD4, C2Y4, XJ406 and XP2-03 promoted the liquid of the deep blue. Therefore, the seven strains were deduced to have the capability of producing polyphenol substance—resveratrol.

### Re-screening the resveratrol-producing endophytes from grapevines

#### TLC

The seven strains of the endophytic fungi showing blue color reaction were tested by thin layer chromatography for qualitative detection of resveratrol. Four kinds of the strains, C2J6, C2Y4, XP2-03 and C2Y6, presented obviously blue spots in the experiment. TLC was often used for the separation and identification of organic compounds. Therefore, the composition of the organic matter could be identified by comparing with the compound of the known structure. When there were the same conditions, such as expansion agent, adsorbent, the thickness of the thin layer plate and temperature, for a compound, the R_f_ value, as a constant, could be used as the basis of qualitative analysis. In this trial, the R_f_ of the strain C2J6 was 0.322, exactly equal to the value of the resveratrol standard, which could conclude preliminarily that the C2J6 could promote the synthesis of the resveratrol. Nevertheless, the R_f_ values of other strains, such as C2Y4, XP2-03, C1G2, XD4, C2Y6 and XJ406, were different from that of the standard sample, which excluded the possibility that these strains produced resveratrol. Therefore, the strain C2J6 was selected as the target strain for the production of resveratrol, which was isolated from the stems of grapevine collected in spring.

#### Ultraviolet wavelength scanning

The result that the resveratrol sample separated and purified was scanned in the range of 200–500 nm was that the maximum absorption wavelength of the resveratrol sample was at 306.5 nm, which was the same as the maximum wavelength of resveratrol standard. Based on the result, the product purified from fermented liquid was just the resveratrol.

#### Liquid chromatography (LC)

LC is the common method of the qualitative and quantitative analysis. Under the condition of the determinate chromatographic system and the operation, each material has a certain retention time. Thus, the unknown material and standard substance can be notarized preliminarily the same material owe to the similar retention time under the same chromatographic conditions.

**Fig. 1:**
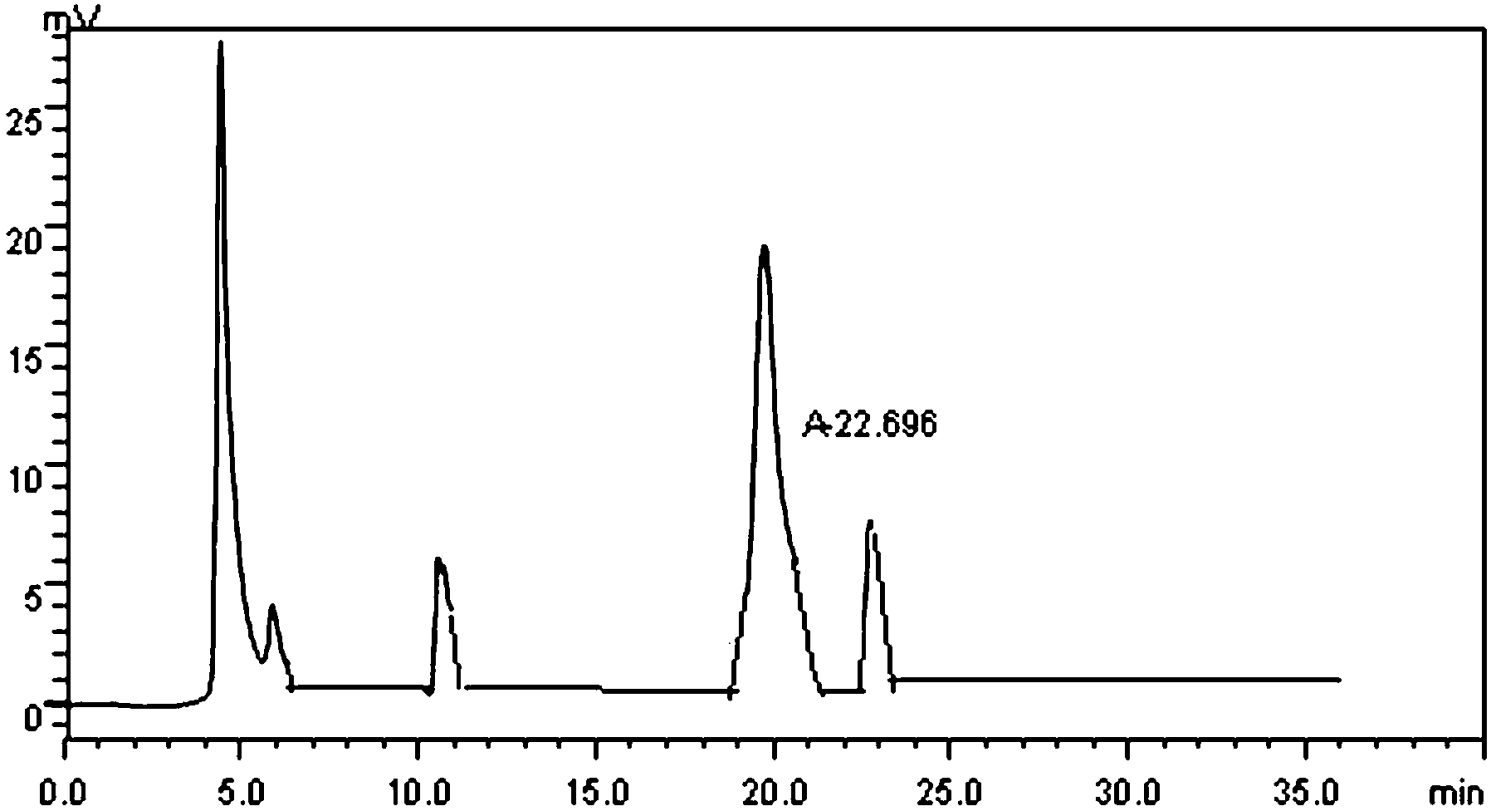
LC profile of the fermentation liquid of the strain C2J6 A, trans-resveratrol

In this experiment, the retention time of the resveratrol, 22.696 min (Fig. 1), in C2J6 fermentation liquor after the separation and purification in the LC chromatogram was the same as that of the standard one, which suggested that the strain C2J6 produced really the resveratrol, and the yield of the resveratrol produced by C2J6 was 1.48 mg L^−1^ according to the standard curve.

### Genetic stability of the resveratrol by C2J6 detection

After the successive culture of five generations for the strain C2J6, the average yield of the resveratrol produced had reached 1.48 mg L^−1^ (Table 2). Statistical analysis showed the stable resveratrol yield, and the strains had also admirable genetic stability producing resveratrol. The resveratrol production of the strain C2J6 was higher than the value (97 μg L^−1^), but lower than that (50–100 μg g^−1^) contained in grape peel reported by Zeng et al. (2012). The difference of the former and the latter was closely related to many factors, such as the grape varieties, growth location, climate, planting technology and fungal infection degree, which resulted in the changes of endophyte microecology and resveratrol yield.

**Table 2:** The stability of production of resveratrol by the strain C2J6

	Number of culture generation					
	1	2	3	4	5	Average
Resveratrol (mg L^−1^)	1.52	1.47	1.45	1.48	1.50	1.48

### Identification of the strain C2J6

#### Growth on the PDA medium

**Fig. 2:**
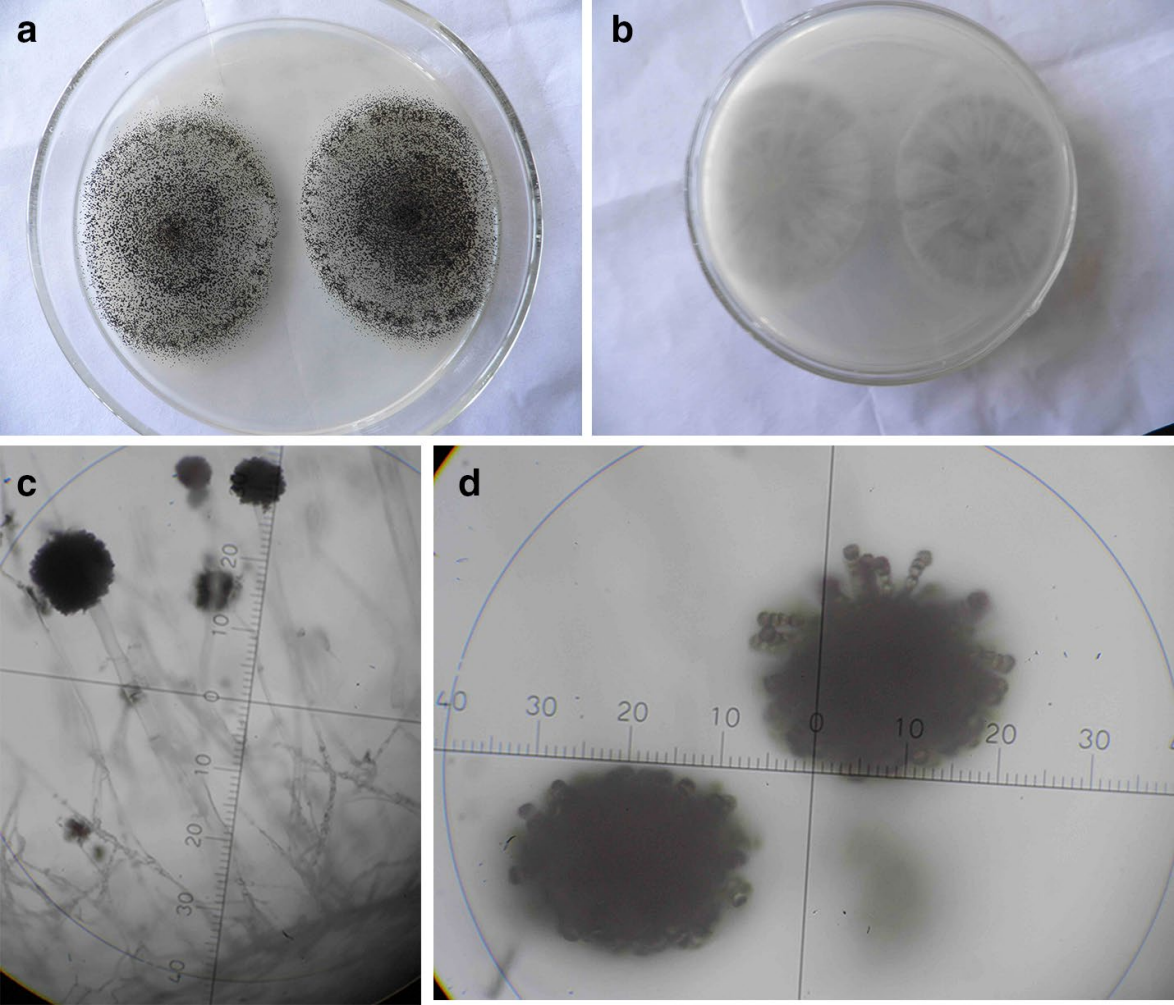
Morphology of the strain C2J6 on Petri dishes and under microscopes. **a**, **b** Morphology of C2J6 on Petri dishes. **c**, **d** Morphology of C2J6 under microscopes

The rounded colony of C2J6 spread rapidly on PDA medium (Fig. 2), but the growth of which was somewhat limited; hypha changed from the white at the beginning to the black at the end in the color and from the short and tight villous to the thick velvet in the shape and density during the gradual process evacuating to the edge of the PDA medium. From the petri dish, the black tophaceous spores distributed densely in the center of the PDA medium, the back, presenting the radial pattern, of which emerged colorlessness or slight central brown.

#### Morphology

Through the microscope, the colorless non-septate hyphae of the strain C2J6 was observed, which extended a lot of the inflated branches without rhizoid and small stalks at the top, while the erect sporangiophore and round mature spores, looked like a string of “conidia”, arranged in a chain (Fig. 2).

**Fig. 3:**
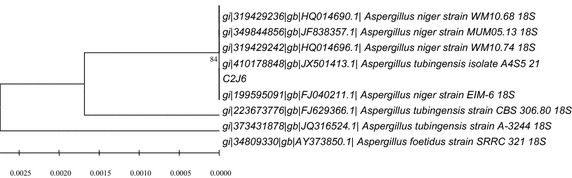
Phylogenetic tree of the strain C2J6 based on 26S rDNA gene sequences

All in all, by means of the colony morphology and microscopic morphology, combined with the manual of fungi identification and sequencing 26S rDNA gene and phylogenetic analysis (Altschul et al. 1990), C2J6 was preliminarily identified as *Aspergillus niger* (Fig. 3).

## Conclusions

From the trial, C2J6, which had stable genetic properties producing resveratrol highly, were screened successively by the methods of thin-layer chromatography, ultra violet wavelength scanning and liquid chromatography, and identified finally as *A. niger* by 26S rDNA-ITS sequence analysis. The mean production of resveratrol from the strain C2J6 could reach 1.48 mg L^−1^ which attested the natural ability of the endogenous bacteria producing the same or similar bioactive substances as the host plants.
